# An Adjuvanted Inactivated SARS-CoV-2 Microparticulate Vaccine Delivered Using Microneedles Induces a Robust Immune Response in Vaccinated Mice

**DOI:** 10.3390/pharmaceutics15030895

**Published:** 2023-03-09

**Authors:** Sharon Vijayanand, Smital Patil, Ipshita Menon, Keegan Braz Gomes, Akanksha Kale, Priyal Bagwe, Mohammad N. Uddin, Susu M. Zughaier, Martin J. D’Souza

**Affiliations:** 1 Vaccine Nanotechnology Laboratory, Center for Drug Delivery and Research, College of Pharmacy, Mercer University, Atlanta, GA 30341, USA; 2 College of Medicine, QU Health, Qatar University, Doha P.O. Box 2713, Qatar

**Keywords:** microneedles, microparticles, SARS-CoV-2, immunogenicity, cytotoxicity, antibody response, T-cell response

## Abstract

SARS-CoV-2, the causal agent of COVID-19, is a contagious respiratory virus that frequently mutates, giving rise to variant strains and leading to reduced vaccine efficacy against the variants. Frequent vaccination against the emerging variants may be necessary; thus, an efficient vaccination system is needed. A microneedle (MN) vaccine delivery system is non-invasive, patient-friendly, and can be self-administered. Here, we tested the immune response produced by an adjuvanted inactivated SARS-CoV-2 microparticulate vaccine administered via the transdermal route using a dissolving MN. The inactivated SARS-CoV-2 vaccine antigen and adjuvants (Alhydrogel^®^ and AddaVax™) were encapsulated in poly(lactic-co-glycolic acid) (PLGA) polymer matrices. The resulting MP were approximately 910 nm in size, with a high percentage yield and percent encapsulation efficiency of 90.4%. In vitro, the vaccine MP was non-cytotoxic and increased the immunostimulatory activity measured as nitric oxide release from dendritic cells. The adjuvant MP potentiated the immune response of the vaccine MP in vitro. In vivo, the adjuvanted SARS-CoV-2 MP vaccine induced high levels of IgM, IgG, IgA, IgG1, and IgG2a antibodies and CD4^+^ and CD8^+^ T-cell responses in immunized mice. In conclusion, the adjuvanted inactivated SARS-CoV-2 MP vaccine delivered using MN induced a robust immune response in vaccinated mice.

## 1. Introduction

Severe acute respiratory syndrome coronavirus-2 (SARS-CoV-2) infection causes COVID-19 in humans and has globally affected the health of individuals with over 1,083,2791 deaths in the United States alone (as of 20 December 2022) [1]. Vaccination against SARS-CoV-2 is the most effective tool used to protect against lower respiratory tract infection or severe disease caused by the virus [2]. Currently, several vaccines for COVID-19 have been approved for use in humans [3]. However, through mutations and immune evasion, SARS-CoV-2 and its variants remain a significant global threat [4]. Therefore, much like the flu shot, frequent vaccination against SARS-CoV-2 may be necessary. With routine immunizations, there is a lack of efficiency during mass vaccination, not to mention the burden on healthcare professionals to administer the vaccines. From a patient’s perspective, individuals face several challenges while vaccinating. Individuals suffering from needle phobia refuse to vaccinate; needle injections are often painful and are not desired by older patients, young children, and toddlers. Therefore, an improvised and patient-friendly vaccination system that can maintain vaccine efficacy while being pain-free and efficient for mass vaccination and frequent immunization is highly desirable.

Recently, the microneedle (MN) system for vaccine delivery has gained much attention, as it is a non-invasive approach with the possibility of self-administration [5,6,7]. Such a versatile system for vaccine delivery is suitable for mass vaccination and frequent immunization and will positively influence the global vaccination rate. From an immunological standpoint, MN administration is advantageous, as the epidermal and dermal layers of the skin are enriched with Langerhans’s cells and circulating dendritic cells that can readily recognize the vaccine antigen, thus activating an immune response [8,9,10]. Here, we explore the use of dissolving MN for administering an inactivated microparticulate SARS-CoV-2 vaccine. These dissolving MN are composed of biodegradable polymers, which are very safe for human use and dissolve within 5 min upon application to deliver the vaccine antigen.

When an inactivated virus vaccine is administered to an individual, the virus is presented as a whole to the immune system, resulting in a broader immune response [11]. SARS-CoV-2 consists of several structural proteins, including the spike (S) protein, membrane (M) protein, nucleoprotein (N), and envelope (E) protein [12,13]. Heat inactivation of the virus destroys the viral RNA, thus preventing the virus from replicating in the host cell while conserving the other structural proteins [14,15]. With relevance to SARS-CoV-2, when an inactivated vaccine is administered, the immune system may generate antibodies and cellular responses against the N protein, M protein, and E protein, in addition to the S protein [11,15,16]. Recent studies have shown that the SARS-CoV-2 variants predominantly harbor spike RBD mutations, resulting in reduced vaccine efficacy against the variants [17,18,19]. Therefore, a vaccine targeting different structural proteins may be more efficacious against the emerging variants [20,21,22,23]. However, to generate responses against the other structural proteins, all the proteins must be presented to the immune system without being degraded or destroyed by the tissue fluids upon vaccine administration [24]. Here, we propose a microparticulate vaccine delivery system encapsulating the inactivated SARS-CoV-2 virus in a polymer matrix.

Encapsulating the vaccine antigen in a biodegradable polymer matrix protects it by providing sustained release, thereby preventing antigen degradation by enzymes in the tissue fluids [25,26,27]. Microparticulate vaccines have also been reported to be better taken up by the circulating dendritic cells (DCs), resulting in more significant translocation to immune organs (draining lymph nodes and spleen) and allowing for increased antigen presentation to the T-cells [28,29,30,31]. In contrast to microparticulate antigens, antigens in the suspension form are smaller in size and are less immunogenic. Therefore, they are poorly recognized and are rapidly cleared by the host’ immune system [32]. In the past, we have demonstrated that encapsulating the vaccine antigen in a polymer matrix can enhance the immunostimulatory activity of the antigen [26,33,34,35]. In the present study, the vaccine antigen and adjuvants are encapsulated in polymer matrices using poly(lactic-co-glycolic acid) (PLGA) to form an effective carrier system. Microparticulate vaccines have previously been reported to be stable at room temperature and thus may be favorable during vaccine storage and distribution for mass vaccinations and global immunization [36,37,38,39,40].

Adjuvants have long been used in vaccines to boost the immune response produced by the vaccine antigen [41]. The adjuvants Alhydrogel^®^ and AddaVax™ are approved for use in licensed vaccines and have been documented to enhance the vaccine response [42]. Alhydrogel^®^ forms a depot when injected and recruits APCs to the site of administration, increasing cellular uptake and antigen presentation to T-cells [43,44,45]. AddaVax™ (MF59-like) is a squalene-based nano-emulsion that induces cytokines and chemokines involved in the recruitment, activation, and maturation of APCs [46,47,48]. Previously, we have tested the adjuvants Alhydrogel^®^ and AddaVax™, encapsulated in polymer matrices in a model microparticulate vaccine formulation [35]. We observed that adjuvants and an inactivated model antigen yielded increased antibody levels in vaccinated mice [35]. Therefore, Alhydrogel^®^ and AddaVax™ were used in this study as adjuvants to enhance the immunogenicity of the inactivated SARS-CoV-2 antigen.

Previously, as a proof of concept, we demonstrated that MN administration of an inactivated microparticulate vaccine utilizing canine coronavirus (CCoV) as a model antigen increased antibody levels in vaccinated mice [35]. In this current follow-up study, we have summarized the results of testing an adjuvanted inactivated SARS-CoV-2 MP vaccine administered to mice using quick-dissolving MNs. First, the vaccine and adjuvant MP were formulated, characterized, and assessed in vitro for immunogenicity and cytotoxicity. The vaccine-loaded MN was then prepared and administered to mice via the skin. The MN quickly dissolves to release the vaccine MP, which may then be taken up by the dendritic cells or macrophages in the skin, subsequently activating the host immune responses against the vaccine antigen. The SARS-CoV-2 specific antibody levels and T-cell responses following MN vaccination were evaluated and are reported in this article.

## 2. Materials and Methods

### 2.1. Materials

Sodium hyaluronate (100 kDa) was obtained from Lifecore Biomedical (Chaska, MN, USA). Poly(lactic-co-glycolic acid) (75:25) was procured from Evonik Industries (Essen, Germany). Polyvinyl alcohol (PVA) (Avg Mol Wt. 30,000–70,000), dichloromethane (DCM), trehalose dihydrate, and lipopolysaccharides (LPSs) from Escherichia coli O111:B4 were purchased from Sigma-Aldrich (St. Louis, MO, USA). The heat-inactivated SARS-CoV-2 antigen was obtained from BEI Resources (NIAID, NIH: Heat Inactivated, SARS-Related Coronavirus 2, Isolate USA-WA1/2020, NR-52286). Alhydrogel^®^ and AddaVax™ were purchased from InvivoGen (San Diego, CA, USA). Pierce Micro BCA™ Assay Kit was obtained from Thermo Fisher Scientific (Waltham, MA, USA). The 8 × 8 array poly dimethyl siloxane (PDMS) MN templates were obtained from Micropoint Technologies (Singapore). Fetal bovine serum (FBS), Dulbecco’s Modified Eagle’s Medium (DMEM), non-essential amino acids, and penicillin/streptomycin were procured from American Type Culture Collection (ATCC) (Manassas, VA, USA). Murine dendritic cells (DCs) were a gift from Kenneth L. Rock at the Dana-Farber Cancer Institute, Inc. (Boston, MA, USA). Six- to eight-week-old Swiss Webster mice were procured from Charles River Laboratories (Wilmington, MA, USA). HRP-tagged secondary goat anti-mouse antibodies IgG, IgM, IgA, IgG1, and IgG2a, were procured from Invitrogen (Rockford, IL, USA). Allophycocyanin (APC)-labeled anti-mouse CD4 and fluorescein isothiocyanate (FITC)-labeled anti-mouse CD8 antibodies were obtained from Invitrogen™, Thermofisher Scientific (Waltham, MA, USA).

### 2.2. Methods

#### 2.2.1. Preparation and Characterization of Microparticles (MP)

The heat-inactivated SARS-CoV-2 (iSARS-CoV-2) vaccine MP and adjuvant MP (Alhydrogel^®^ and AddaVax™) were prepared using a double emulsion method with solvent evaporation as described previously [9,33,34,35]. First, the inactivated SARS-CoV-2 antigen (1% loading) in a pH 7.4 phosphate buffer was added to PLGA in DCM solution (2% *w*/*v*) and probe-homogenized at 17,000 RPM using a 30 s on/30 s off cycle for 2 min (primary emulsion). Next, the primary emulsion was probe-homogenized with the PVA solution in deionized water (0.1% *w*/*v*) for 2 min at 17,000 RPM (double emulsion). The final emulsion was stirred at 500 RPM for 5 h to remove the residual DCM via solvent evaporation. The excess PVA was removed by washing with deionized water followed by centrifugation at 17,000 RPM for 10 min. The MP was resuspended with 1 mL of trehalose solution (2% *w*/*v*) to serve as a cryoprotectant. The Alhydrogel^®^ MP (10% loading) and AddaVax™ (5% loading) MP were prepared similarly by substituting the antigen with the adjuvant in the primary emulsion. The final vaccine MP and adjuvant MP formulations were freeze-dried to obtain the dry product. The percent recovery yield of the lyophilized product was calculated using the following formula as described previously [35,49,50].

(1) $$Percent\;Recovery\;yield=\frac{Weight\;of\;lyophilized\;MP*100}{Weight\;of\;all\;ingredients\;in\;the\;formulation}$$

The MP were observed under the scanning electron microscope and characterized for size and shape. The MP size and the surface charge were measured using a Malvern Zetasizer Nano ZS (Malvern Panalytical Ltd., Worcestershire, UK), as described previously [35]. The encapsulation efficiency (EE) of the inactivated SARS-CoV-2 antigen in the MP was assessed as described previously [33,34,35,51]. Briefly, DCM was added to 5 mg of the vaccine MP to dissolve the PLGA matrix. The solution was centrifuged to concentrate the antigen into a pellet. The supernatant was discarded, and the concentrated antigen pellet was placed in a vacuum chamber for 30 min to remove the residual DCM by evaporation. The antigen was resuspended using 1 mL PBS and analyzed using a micro-Bicinchoninic acid (BCA) assay per the manufacturer’s instructions. The concentration per ml (conc/mL) was determined by plotting a standard curve. The percentage encapsulation efficiency (% EE) was calculated using the following formula, as described previously [34,35,51].

(2) $$\%EE=\frac{Practical\;concentration\;of\;antigen\;in\;5mg\;of\;MP\times 100}{Theoretical\;concentration\;of\;antigen\;in\;5mg\;of\;MP}$$

#### 2.2.2. Evaluating the In Vitro Immunostimulatory Activity of the Vaccine MP

The in vitro immunogenicity of the MP was assessed by measuring the nitrite released by the DCs exposed to the different MP groups. As described previously, the nitrite accumulated consequent to nitric oxide production by the DCs was quantified using Griess’ nitrite assay [26,33,34,35]. Briefly, in a 96-well plate, murine DCs were plated at a density of 1 × 10^4^ cells/well. The cells were then pulsed with a calculated amount of antigen MP and adjuvant MP and were incubated for 24 h at 37 °C. The groups tested and their corresponding dose/wells are listed as follows: lipopolysaccharide (LPS) from *Escherichia coli* (positive control) (2 μg), no treatment (negative control), blank MP, Alhydrogel^®^ MP (3 μg), AddaVax™ MP (0.5 μg), inactivated SARS-CoV-2 (iSARS-CoV-2) suspension (2 μg), iSARS-CoV-2 MP vaccine (2 μg), adjuvanted iSARS-CoV-2 MP vaccine (iSARS-CoV-2 (2 μg) + Alhydrogel^®^ (3 μg) + AddaVax™ (0.5 μg)). After the 24 h incubation, the supernatants (50 μL/well) were transferred to a fresh 96-well plate. To each well, 50 μL of sulfanilamide (1%) in phosphoric acid (5%) was added and incubated for 5–10 min, protected from light at room temperature. Next, 50 μL of NED (0.1%) (*N*-1-naphthyl ethylenediamine dihydrochloride) solution in deionized water was added and kept at room temperature for 5–10 min, protected from light. The appearance of a purple/magenta color indicated nitrite release. The absorbance (540 nm) was read using a plate reader (Bio Tek Synergy, BIO-TEK Instruments, Winooski, VT, USA). A sodium nitrite standard curve was plotted from which the nitrite content was quantified.

#### 2.2.3. Determining the In Vitro Cytotoxicity of the Vaccine MP

The cytotoxicity of the inactivated SARS-CoV-2 MP to DCs was assessed in vitro using an MTT assay (3-(4,5-Dimethylthiazol-2-yl)-2,5-diphenyltetrazolium bromide) described previously [33,34,35]. The cytotoxicity of the adjuvant MP (Alhydrogel^®^ MP and AddaVax™ MP) has been previously tested and was found to be non-cytotoxic in varying concentrations [35]. To assess the cytotoxicity of the inactivated SARS-CoV-2 MP, DCs were plated at a cell density of 1 × 10^4^ cells/well in a 96-well plate. Then, two-fold serial dilutions of the inactivated SARS-CoV-2 MP (31.25 ug/mL to 500 µg/mL) were prepared in cDMEM (DMEM high glucose medium with 2 mM L-glut, 1% penicillin–streptomycin, sodium pyruvate, 10% FBS). The diluted MP suspensions were added in triplicate to each well and incubated for 24 h at 37 °C. Cells that received no treatment were used as the positive control, and cells treated with 50 μL of DMSO were used as the negative control. After the incubation period, the media containing the suspended MP were removed gently using a pipette. Next, 10 μL MTT reagent (5 mg/mL) was added to every well, and the volume was made up to 100 μL/well with cDMEM. The plate was kept for incubation at 37 °C for 4 h, protected from light. Following incubation, 100 μL/well of DMSO was added, and the absorbance (570 nm) was measured using a plate reader.

#### 2.2.4. Preparation of Vaccine-Loaded MNs

As described previously, a spin casting method was used to prepare the vaccine-loaded quick-dissolving MN [9,35]. Then, 10% *w*/*v* sodium hyaluronate and 5% *w*/*v* trehalose in deionized water constituted the MN gel base. Then, the vaccine MP and the adjuvants MP required for each patch were weighed and dispersed into the MN gel base. Then, 25 mg of gel was added to each pre-weighed PDMS MN mold and centrifuged at 4000 rpm for 15 min at 15 °C to form the MN. The MN molds were kept overnight for drying, and 10% HA gel was added as backing the following day. The dried MN were removed from the molds and observed under the scanning electron microscope for its physical appearance.

#### 2.2.5. In Vivo Immunization Procedure and Dosing Regimen

**Figure 1:**
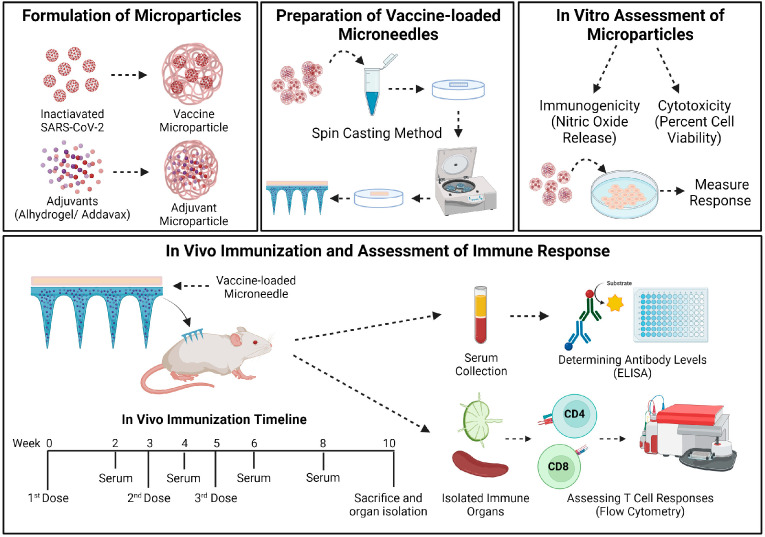
Schematics of methodology used in this study. The vaccine MP and adjuvant MP were formulated and characterized. The vaccine-loaded quick-dissolving MN were prepared using a spin casting method. The MP were assessed in vitro for their immunogenicity and cytotoxicity. The vaccine-loaded MN were administered to mice following which both antibody levels and cellular responses were assessed and reported. The mice received three doses of the adjuvanted inactivated SARS-CoV-2 MP MN vaccine at weeks 0, 3, and 5. The mice were sacrificed at week 10, and the immune organs (spleen and lymph node) were isolated and processed into single-cell suspensions to analyze T-cell responses. The image was created using BioRender.com (accessed on 7 March 2023).

The vaccine efficacy of the adjuvanted inactivated SARS-CoV-2 MP vaccine administered using dissolving MN was tested in vivo in 6–8-week-old male Swiss Webster (CFW) mice, *n* = 4. The mice were immunized via the skin with the vaccine-loaded MN patches. The testing was performed as per the approved Mercer University IACUC protocol (animal protocol #A2004006). The antigen dose was 20 μg/mouse of inactivated SARS-CoV-2, the Alhydrogel^®^ dose was 30 μg/mouse, and the AddaVax™ dose was 5 μg/mouse. The animals were divided into two groups: a no treatment control group and the adjuvanted inactivated SARS-CoV-2 vaccine MP group. The vaccine group received the adjuvanted MP vaccine using the dissolving MNs via the transdermal route. Before immunization, a 2 × 2 cm patch of the fur was removed from the back of the anesthetized mice (inhalational Isoflurane) using a depilatory cream for ease of MN application. The animals received three doses of the vaccine at weeks 0, 3, and 5. The mice were bled bi-weekly, and the serum was collected for determining the antibody levels. The animals were sacrificed at week 10, and their immune organs, including the spleen and lymph node, were isolated and processed into single-cell suspensions to analyze T-cell responses (Figure 1).

#### 2.2.6. Determining the Serum Antibody Levels in Immunized Mice

The mice were bled bi-weekly, and the serum was isolated to evaluate the SARS-CoV-2-specific antibody responses. An enzyme-linked immunosorbent assay was used to determine the serum IgM, IgG, IgA, IgG1, and IgG2a levels described previously [35]. For this purpose, high-binding 96-well plates (MICROLON^®^, 96-well plate, High binding, Greiner bio one) were coated with 50 μL/well of the inactivated SARS-CoV-2 antigen (0.2 μg/well) in a pH 9.6 carbonate buffer solution. The coated plates were kept overnight at 4 °C to facilitate attachment of the antigen. Following incubation, plates were washed with 200 μL of 0.01% Tween-20 PBS (T-PBS) solution and blocked with 50 μL/well of 3% Bovine Serum Albumin (BSA) in T-PBS (blocking solution) for 3 h at 37 °C. The plates were rewashed, and the diluted serum sample (50 μL/well) was added to the wells and incubated overnight at 4 °C. The plates were washed again, and 50 μL/well of the HRP-tagged secondary goat anti-mouse IgM, IgG, IgA, IgG1, and IgG2a antibodies (1:2000 to 1:4000) were added and incubated at 37 °C for 90 min. Next, the plates were washed, and 50 μL/well of the TMB (3,3′,5,5″-tetramethyl benzidine) substrate reagent (BD OptEIA™, BD Biosciences, San Jose, CA, USA) was added to each well and kept at room temperature for 10 min. The reaction was stopped by adding 50 μL of 0.3 M H_2_SO_4_ to each well. The absorbance was read at 450 nm using a plate reader.

#### 2.2.7. Evaluating the T-Cell Responses in Immunized Mice

The mice were sacrificed during week 10, and spleen and lymph nodes (inguinal and brachial) were isolated and processed into single-cell suspensions as described previously [26,52]. The red blood cells (RBCs) in the spleen were lysed by adding ammonium chloride potassium (ACK) lysis buffer. The cells were centrifuged to remove the lysed RBCs at 1200 rpm for 10 min, and the splenocytes were resuspended in DMEM containing 70% fetal bovine serum (FBS). Then, 5% *v*/*v* DMSO was added to the cells as a cryoprotectant, frozen at −80 °C. The percentage (%) expression of CD4^+^ and CD8^+^ T-cells in the lymph nodes and spleen cells was evaluated using a flow cytometer. First, the cell suspensions were quickly thawed and centrifuged at 1200 rpm to remove the media and DMSO. The cells were resuspended using fresh DMEM and stimulated with 5 ng/mL IL-2 overnight. The following day, the cells were centrifuged at 1200 rpm to remove the IL-2, and the cells were resuspended using fresh DMEM. The cells were then stimulated with 5 μg/mL of the inactivated SARS-CoV-2 antigen overnight. As the mice were not challenged with the live SARS-CoV-2 virus, the splenocytes and lymphocytes were stimulated in vitro with the vaccine antigen to test the specificity of the CD4+ and CD8+ T-cells toward SARS-CoV-2. Following incubation with the antigen, the cells were centrifuged at 1200 rpm to form a pellet. The cells were resuspended in 100 μL of the marker solution containing APC-labeled anti-mouse CD4 and FITC-labeled anti-mouse CD8 antibodies in PBS. The cells were incubated for 1 h on ice, protected from light, and gently vortexed every 15 min. Following incubation, the cells were washed 3 times and analyzed using flow cytometry.

#### 2.2.8. Statistical Analysis

Statistical analysis was performed using GraphPad Prism 9.2.0 software (GraphPad Software, San Diego, CA, USA). One-way ANOVA was used for normally distributed data with independent groups. Two-way ANOVA was used for dependent groups. A post hoc Sidak’s test was used for multiple comparisons between the two groups. For multiple comparisons between three or more groups, a post hoc Tukey test (to compare between means) or post hoc Dunnett test (to compare means to control) was used. The following *p* values were used, *p* > 0.05 (ns—non-significant), *p* ≤ 0.05 (*), *p* ≤ 0.01 (**), *p* ≤ 0.001 (***), and *p* ≤ 0.001 (****). A *p* value < 0.05 is considered statistically significant. Data are expressed as mean ± standard error mean (SEM).

## 3. Results

### 3.1. Characterization of Vaccine MP and MN

**Figure 2:**
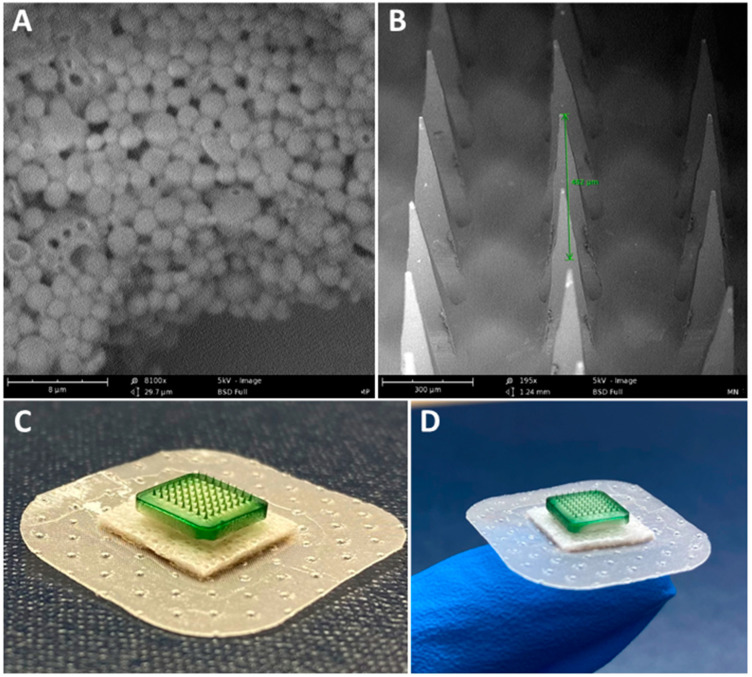
Characterization of vaccine MP and vaccine-loaded MN. (**A**) Scanning electron microscope image of the vaccine MP (magnification—8100×). The vaccine MP was spherical with smooth surfaces. (**B**) Scanning electron microscope image of an MN patch (magnification—195×). The MN was approximately 482 μm in length. (**C**,**D**) Indocyanine green (ICG)-loaded MN band-aid patch. ICG was used for better visualization of MN.

The percentage yield of the inactivated SARS-CoV-2 MP was 83%, with an encapsulation efficiency of 90.4%, size of approximately 910 nm, and surface charge of −23.11 mV. The percentage yield of the Alhydrogel^®^ MP was 93%, with a size of roughly 1.3 μm and a surface charge of 12 mV. The percentage yield of the AddaVax™ MP was 90.5%, with an approximate size of 1.2 μm and a surface charge of −12.5 mV. Scanning electron microscope images show that the inactivated SARS-CoV-2 MP were spherical with smooth surfaces (Figure 2A). The dissolving MNs were also observed under the scanning electron microscope. The images show the formation of sharp needles with a length of approximately 482 μm (Figure 2B). A detailed characterization analysis of dissolving MNs was previously published by our group [9].

### 3.2. Vaccine MP Show Enhanced Immunostimulatory Activity In Vitro

**Figure 3:**
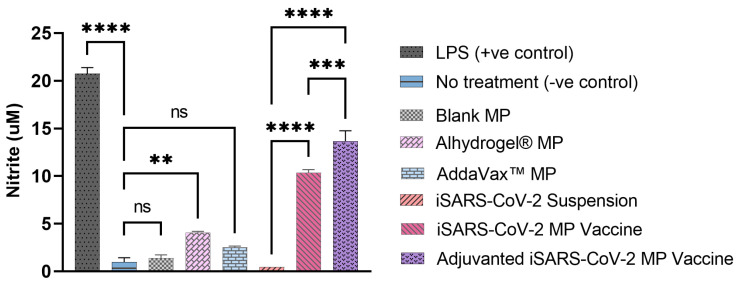
Nitric oxide (NO) released by the DCs upon exposure to the different treatment groups. The cell density was adjusted to 1 × 10^4^ cells/well and treated with the following groups for 24 h: lipopolysaccharide (LPS) (2 μg) (+ve control), no treatment (−ve control), blank MP, Alhydrogel^®^ MP (3 μg), AddaVax™ (0.5 μg), iSARS-CoV-2 suspension (2 μg), iSARS-CoV-2 MP vaccine (2 μg), adjuvanted iSARS-CoV-2 MP vaccine (iSARS-CoV-2 (2 μg) + Alhydrogel^®^ (3 μg) + AddaVax™ (0.5 μg)). The nitrite released in the supernatant was assessed using the Griess’ nitrite assay method. Data expressed as mean ± SEM, *n* = 3, one-way ANOVA test, post hoc Dunnett’s multiple comparisons test, ns: non-significant, ** *p* ≤ 0.01, *** *p* ≤ 0.001, and **** *p* ≤ 0.0001.

The immune recognition or biologic activity of the formulated iSARS-CoV2 MP by DC was assessed using an in vitro assay. The nitric oxide (NO) released by the DCs pulsed with vaccine MP was measured and quantified. The blank MP and the AddaVax™ MP did not release significant levels of NO compared to the cells that received no treatment or LPS-treated DC (Figure 3). Alhydrogel^®^ MP produced significant levels of NO compared to the no treatment group. The cells that received the inactivated SARS-CoV-2 antigen suspension had negligible NO. However, the cells that received the inactivated SARS-CoV-2 MP vaccine produced significantly high levels of NO compared to the antigen in the suspension form. Further, adding adjuvants Alhydrogel^®^ and AddaVax™ significantly increased the NO release and resulted in a significant difference compared to the unadjuvanted MP group (Figure 3).

### 3.3. Vaccine MP Are Non-Cytotoxic to DCs

**Figure 4:**
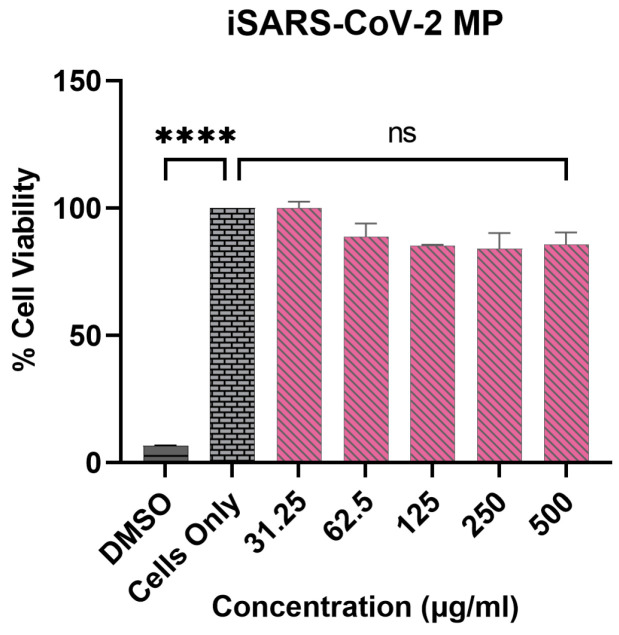
Percent cell viability of DCs pulsed with iSARS-CoV-2 MP. The cell density was adjusted to 1 × 10^4^ cells/well. Two-fold serial dilutions of the iSARS-CoV-2 MP in cDMEM (concentration range: 31.25 to 500 ug/mL) were added to every well at a volume of 100 μL/well and incubated for 24 h. Cell only and cells treated with DMSO (50 μL) were used as positive and negative controls, respectively. Data expressed as mean ± SEM, *n* = 3, one-way ANOVA test, post hoc Dunnett’s multiple comparison test, ns: non-significant, **** *p* ≤ 0.0001.

The cytotoxicity of the inactivated SARS-CoV-2 MP was tested in vitro using the MTT assay. The cytotoxicity of the adjuvant MP was previously assessed and reported to be non-toxic to cells in specific concentrations [35]. Consistent with our previously published data, the inactivated SARS-CoV-2 MP vaccine was also found to be non-cytotoxic and resulted in no significant cell death up to a concentration of 500 μg/mL. Dimethyl sulfoxide (DMSO) was used as a positive control and resulted in a significant decrease in the percent cell viability compared to the cells only control (Figure 4).

### 3.4. Adjuvanted Vaccine Increased Antibody Levels in Immunized Mice

**Figure 5:**
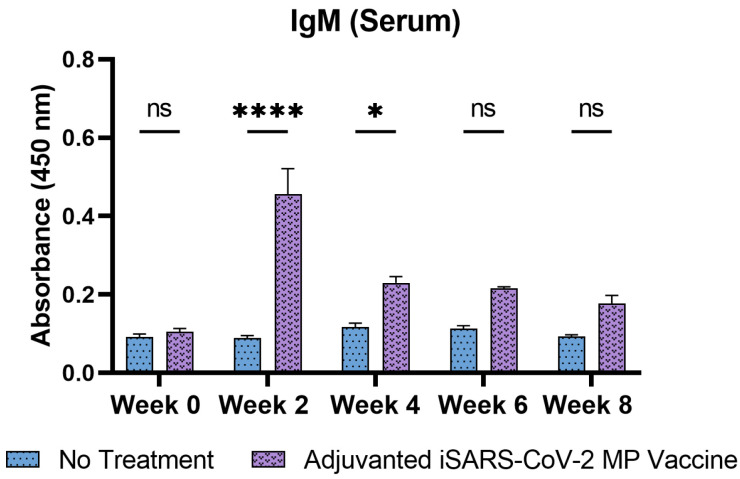
Serum IgM antibody levels in vaccinated mice. Responses obtained are compared to the no treatment (control). Data expressed as mean ± SEM, *n* = 4, two-way ANOVA, post hoc Sidak’s multiple comparisons test. ns: non-significant, * *p* ≤ 0.05, **** *p* ≤ 0.0001.

Following immunization of mice with the vaccine MN, the antibody levels in the mice sera were assessed using ELISA. The SARS-CoV-2-specific IgM, IgG, IgA, IgG1, and IgG2a antibody levels were detected and quantified. The serum IgM levels peaked during week 2 following the prime dose and subsequently decreased during the later weeks (Figure 5).

**Figure 6:**
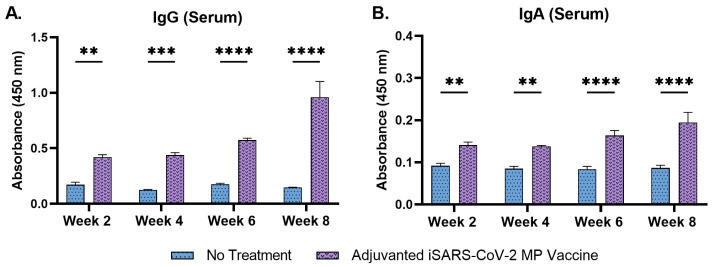
Serum IgG and serum IgA antibody levels in vaccinated mice. (**A**) Total IgG in vaccinated mice. (**B**) Total IgA in vaccinated mice. Responses obtained are compared to the no treatment (control). Data expressed as mean ± SEM, *n* = 4, two-way ANOVA, post hoc Sidak’s multiple comparisons test. ** *p* ≤ 0.01, *** *p* ≤ 0.001, **** *p* ≤ 0.0001.

The serum IgG levels increased following the prime dose and remained significantly high until week 8 (Figure 6A). The serum IgA levels increased considerably following the prime dose and remained high until week 8 (Figure 6B).

**Figure 7:**
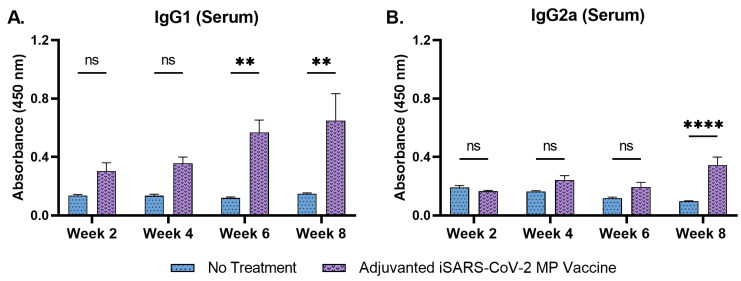
Serum IgG1 and serum IgG2a antibody levels in vaccinated mice. (**A**) Serum IgG1 levels in vaccinated mice. (**B**) Serum IgG2a levels in vaccinated mice. Responses obtained are compared to no treatment (control). Data expressed as mean ± SEM, *n* = 4, two-way ANOVA, post hoc Sidak’s multiple comparisons test. ns: non-significant, ** *p* ≤ 0.01, **** *p* ≤ 0.0001.

IgG subtyping revealed that the serum IgG1 levels increased significantly during weeks 6 and 8 following the second booster dose, administered at week 5 (Figure 7A). Serum IgG2a levels increased significantly at week 8 (Figure 7B). The data show that the adjuvanted iSARS-CoV2 MP delivered by microneedles induced a robust antibody response against the vaccine antigen.

### 3.5. Adjuvanted Vaccine Produced T-Cell Responses in Immunized Mice

**Figure 8:**
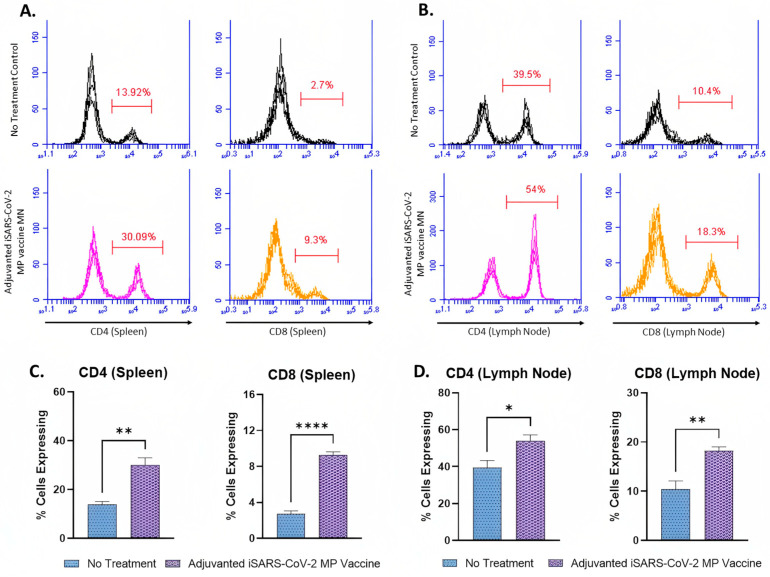
CD4^+^ and CD8^+^ T-cells in the splenocytes and lymphocytes of immunized mice. Responses obtained are compared to no treatment (control). Data expressed as mean ± SEM, *n* = 4, Student’s *t* test. * *p* ≤ 0.05, ** *p* ≤ 0.01, **** *p* ≤ 0.0001. (**A**,**C**) Percentage of CD+ and CD8+ T-cells in the splenocytes of the vaccinated mice. (**B**,**D**) Percentage of CD+ and CD8+ T-cells in the lymphocytes of the vaccinated mice.

The expression of CD4 and CD8 molecules on the surface of the activated T-cells in the splenocytes and lymphocytes of the vaccinated mice was assessed using flow cytometry analysis. The percentages of CD4^+^ (30.09%) and CD8^+^ (9.3%) T-cells in the splenocytes of the vaccinated mice were higher compared to the percentage of CD4^+^ (13.92%) and CD8^+^ (9.3%) T-cells of the no treatment control group (Figure 8A,C). The percentage of T-cells expressing CD4 molecules (30.09%) was higher than that of T-cells expressing CD8 molecules (9.3%) in the splenocytes of the vaccinated mice (Figure 8A). The percentage of CD4^+^ (54%) and CD8^+^ (18.3%) T-cells in the lymph nodes of the vaccinated mice were higher compared to the percentage of CD4^+^ (39.5%) and CD8^+^ (10.4%) T-cells of the no treatment control group (Figure 8B,D). Similar to the results obtained from the splenocytes, the percentage of T-cells expressing CD4 molecules (54%) was higher than that of T-cells expressing CD8 molecules (18.3%) (Figure 8B).

## 4. Discussion

Here, we formulated and characterized an adjuvanted inactivated SARS-CoV-2 microparticulate vaccine and evaluated the immune response generated when administered to mice using MNs. The vaccine was evaluated in vitro for its immunogenicity and cytotoxicity to DC. It was tested in vivo for its ability to induce SARS-CoV-2-specific antibodies and T-cell responses in vaccinated mice.

The inactivated SARS-CoV-2 MP vaccine, Alhydrogel^®^ MP, and AddaVax™ MP were formulated using PLGA as the polymer matrix for encapsulating the antigen or adjuvant. Encapsulating the antigen in a polymer matrix protects the antigen [24]. It has been previously shown to reduce antigen degradation by enzymes present in the tissue fluids while also increasing cellular uptake of the antigen due to its increased size [28,31,40]. The MP product yield using the double emulsion method was greater than 80% with minimal loss during processing. The particles were spherical, with an optimum size ranging from 900 to 1300 nm. MP of 1 to 3 μm size are reported to be efficiently recognized and engulfed by circulating APCs [27]. The charges of the vaccine and adjuvant MP were positive or negative depending on the encapsulated material and were consistent with previously published results [35]. Typically, colloidal suspensions are stable when MP have a high positive or negative charge that prevent agglomeration when the particles are suspended [53]. The %EE of the inactivated SARS-CoV-2 antigen in the PLGA MP was 90.4%, indicating that the double emulsion method’s formulation process results in an effectively loaded carrier system.

Particulate vaccines are more immunogenic than antigens in suspension and are thus capable of producing a more robust immune response [32]. Previously, we have shown that the double emulsion method of encapsulating vaccine antigens in a polymer matrix increases the immunogenicity, cellular uptake, and antigen presentation compared to the antigen suspension [26,33,34,35]. We tested the in vitro immunogenicity of the MP using a Griess’ nitrite assay. APCs such as DCs and macrophages release NO, a non-specific innate immune marker when encountered with an invading pathogen [54,55]. NO is crucial in recruiting APCs and releasing cytokines upon infection, triggering the host cells to produce a robust immune response against the invading pathogen [56,57]. Consistent with our previously published results, we observed that the particulate vaccine was better able to stimulate DCs than the inactivated SARS-CoV-2 antigen suspension [35].

The adjuvant MP was evaluated individually to assess for NO release in pulsed DCs. Alhydrogel^®^ MP (3 μg) induced higher NO levels than the AddaVax™ MP (0.5 μg) due to the differences in the adjuvant concentrations tested. Previously, we tested the in vitro cytotoxicity of varying concentrations of the adjuvant MP (Alhydrogel^®^ and AddaVax™) in DCs [35]. The metabolically active cells (live cells) can reduce MTT salts to purple formazan crystals, which were used to assess the cytotoxicity of the vaccine MP. We observed that increased concentrations of AddaVax™ MP (>0.625 μg) resulted in significant cytotoxicity, whereas Alhydrogel^®^ MP was non-cytotoxic even at higher concentrations [35]. Since it is essential for the vaccine MP and adjuvant MP to be non-cytotoxic to the target cells, we evaluated a lower dose of the AddaVax™ MP for its in vitro immunogenicity, which explains the negligible NO release from the DCs pulsed with just the AddaVax™ MP. However, the Alhydrogel^®^ MP and AddaVax™ MP, when combined with the SARS-CoV-2 vaccine MP, significantly increased the immunogenicity of the vaccine MP. We further tested the percent viability of the cells exposed to the SARS-CoV-2 vaccine MP. The cytotoxicity study suggested that the vaccine MP was non-cytotoxic at a wide range of concentrations (31.25 to 500 μg/mL). The in vitro studies indicated that the MP vaccine was immunogenic and non-cytotoxic at various doses.

We then evaluated the in vivo immune response produced by the adjuvanted inactivated SARS-CoV-2 MP vaccine administered to mice using quick-dissolving MN. Vaccine administration via the skin is promising, as the immune-rich layers of the skin contain several APCs, such as Langerhans cells and DCs, that can promptly recognize and engulf the microparticulate vaccine, thereby triggering robust immune response activation [5,6,58]. After vaccine administration, the mice were bled biweekly, and the serum was evaluated for antigen-specific antibody levels. A functional vaccine needs to generate antibodies specific to the vaccine antigen, as it can recognize and bind to the invading pathogen, thereby preventing it from infecting the host.

It has been reported that IgM appears during the early weeks of vaccination and undergoes isotype class switching to IgG and IgA antibodies [59]. Consistent with the published literature, our vaccine produced high IgM levels during week 2, which decreased significantly in the following weeks. The serum IgG and the serum IgA antibody levels increased following the prime dose and remained significantly high up to week 10. Serum IgA and IgG have been reported to play a critical role in SARS-CoV-2 binding and neutralization [60,61]. MN administration of the adjuvanted iSARS-CoV-2 MP vaccine increased IgG and IgA antibody levels. Here, the antibodies were evaluated in terms of their capacity to bind to the inactivated virus efficiently, as IgG and IgA are the primary humoral antibodies conferencing protection in tissues and at mucosal surfaces. The vaccine’s efficacy will be established by assessing the neutralization capacity of the IgG and IgA antibodies as a follow-up in our future studies. IgG subtyping was performed to characterize and understand the type of Th-mediated (Th1 and Th2) responses produced upon vaccination. Typically, a Th2-type response is associated with increased IgG1 levels [62,63]. Th2-type responses signal the helper T-cells, which play a vital role in activating host immune responses against the invading pathogen [62]. The IgG2a levels are often associated with Th-1 type response [64]. Such a response will trigger cellular responses, predominantly signaling the cytotoxic T-cells and APCs, causing their migration to the invading pathogen [65]. We observed that MN vaccination increased serum IgG1 levels during weeks 6 and 8 following the second booster dose administration at week 5. The serum IgG2a levels increased only during week 8. The IgG1 antibody levels were higher than the IgG2a antibody levels, suggesting that the immune responses produced by the vaccine may be predominantly of Th-2 type.

To further corroborate the results obtained from the antibody assessment, we characterized the cell-mediated immune responses produced upon vaccination. The percentage of CD4+ and CD8+ T-cells in the splenocytes and lymphocytes was assessed using flow cytometry analysis. The Results section shows that the vaccine induced significant levels of SARS-CoV-2-specific CD4+ and CD8+ T-cells. Similar to the results obtained from the antibody analysis, the percentage of CD4+ T-cells was higher than that of CD8+ T-cells. The increased percentage of CD4+ T-cells confirms that the Th-2 type responses are higher than the Th-1 type responses.

Further, CD4+ helper T-cells play a crucial role in the activation and maturation of plasma B-cells responsible for antibody production [63]. Our results suggest that the increased antibody levels may be attributed to the increased CD4+ T-cells. Although the CD4+ T-cells are higher in number, the vaccine is not devoid of CD8+ T-cells. MN vaccine administration produced significant CD4+ and CD8+ T-cells, and both responses are necessary for an efficacious vaccine.

In future work and follow-up studies, we will evaluate the vaccine’s efficacy by assessing the neutralizing antibody titers using a pseudovirus neutralization assay (PVNA). We will further test the durability of the antibody responses generated by the microparticulate method of vaccine formulation. The microparticulate vaccine will also be evaluated for memory responses by assessing the memory markers in the isolated B-cells and T-cells.

## 5. Conclusions

In summary, the adjuvanted inactivated SARS-CoV-2 MP vaccine was formulated and characterized. The microparticulate vaccine was non-cytotoxic and more immunogenic than the soluble antigen suspension in vitro. The vaccine MP delivered via microneedles effectively induced humoral and cell-mediated immune responses in vaccinated mice. When combined with MN administration, the microparticulate method for vaccine delivery can be an effective tool for frequent immunization and mass vaccination. However, like every vaccine, these novel strategies require significant groundwork and validation to be utilized in the marketed formulations.

## Data Availability

Data will be made available on reasonable request.

## References

[1] CDC COVID Data Tracker. Centers for Disease Control and Prevention 2020. https://covid.cdc.gov/covid-data-tracker.

[2] CDC COVID-19 Vaccination. Centers for Disease Control and Prevention 2020. https://www.cdc.gov/coronavirus/2019-ncov/vaccines/effectiveness/why-measure-effectiveness/breakthrough-cases.html.

[3] COVID-19 Vaccines|FDA, n.d. https://www.fda.gov/emergency-preparedness-and-response/coronavirus-disease-2019-covid-19/covid-19-vaccines.

[4] Science Brief: Emerging SARS-CoV-2 Variants|CDC n.d. https://www.cdc.gov/coronavirus/2019-ncov/science/science-briefs/scientific-brief-emerging-variants.html.

[5] Menon I., Bagwe P., Gomes K.B., Bajaj L., Gala R., Uddin M.N., D’souza M.J., Zughaier S.M. (2021). Microneedles: A New Generation Vaccine Delivery System. Micromachines.

[6] Prausnitz M.R., Mikszta J.A., Cormier M., Andrianov A.K. (2009). Microneedle-based vaccines. Curr. Top. Microbiol. Immunol..

[7] Prausnitz M. (2019). A Phase I Study of the Safety, Reactogenicity, Acceptability and Immunogenicity of Inactivated Influenza Vaccine Delivered by Microneedle Patch or by Hypodermic Needle. clinicaltrials.gov.

[8] Kim Y.-C., Prausnitz M.R. (2011). Enabling skin vaccination using new delivery technologies. Drug Deliv. Transl. Res..

[9] Braz Gomes K., D’Souza B., Vijayanand S., Menon I., D’Souza M.J. (2022). A dual-delivery platform for vaccination using antigen-loaded nanoparticles in dissolving microneedles. Int. J. Pharm..

[10] Aldawood F.K., Andar A., Desai S. (2021). A Comprehensive Review of Microneedles: Types, Materials, Processes, Characterizations and Applications. Polymers.

[11] Li Z., Xiang T., Liang B., Deng H., Wang H., Feng X., Quan X., Wang X., Li S., Lu S. (2021). Characterization of SARS-CoV-2-Specific Humoral and Cellular Immune Responses Induced by Inactivated COVID-19 Vaccines in a Real-World Setting. Front. Immunol..

[12] Satarker S., Nampoothiri M. (2020). Structural Proteins in Severe Acute Respiratory Syndrome Coronavirus-2. Arch. Med. Res..

[13] Yadav R., Chaudhary J.K., Jain N., Chaudhary P.K., Khanra S., Dhamija P., Sharma A., Kumar A., Handu S. (2021). Role of Structural and Non-Structural Proteins and Therapeutic Targets of SARS-CoV-2 for COVID-19. Cells.

[14] Batéjat C., Grassin Q., Manuguerra J.-C., Leclercq I. (2021). Heat inactivation of the severe acute respiratory syndrome coronavirus 2. J. Biosaf. Biosecur..

[15] Li X.N., Huang Y., Wang W., Jing Q.L., Zhang C.H., Qin P.Z., Guan W.J., Gan L., Li Y.L., Liu W.H. (2021). Effectiveness of inactivated SARS-CoV-2 vaccines against the Delta variant infection in Guangzhou: A test-negative case-control real-world study. Emerg. Microbes Infect..

[16] Gao Q., Bao L., Mao H., Wang L., Xu K., Yang M., Li Y., Zhu L., Wang N., Lv Z. (2020). Development of an inactivated vaccine candidate for SARS-CoV-2. Science.

[17] Ding C., He J., Zhang X., Jiang C., Sun Y., Zhang Y., Chen Q., He H., Li W., Xie J. (2021). Crucial Mutations of Spike Protein on SARS-CoV-2 Evolved to Variant Strains Escaping Neutralization of Convalescent Plasmas and RBD-Specific Monoclonal Antibodies. Front. Immunol..

[18] Harvey W.T., Carabelli A.M., Jackson B., Gupta R.K., Thomson E.C., Harrison E.M., Ludden C., Reeve R., Rambaut A., COVID-19 Genomics UK (COG-UK) Consortium (2021). SARS-CoV-2 variants, spike mutations and immune escape. Nat. Rev. Microbiol..

[19] Magazine N., Zhang T., Wu Y., McGee M.C., Veggiani G., Huang W. (2022). Mutations and Evolution of the SARS-CoV-2 Spike Protein. Viruses.

[20] Viral Targets for Vaccines against COVID-19|Nature Reviews Immunology n.d. https://www.nature.com/articles/s41577-020-00480-0.

[21] Fan S., Sun W., Fan L., Wu N., Sun W., Ma H., Chen S., Li Z., Li Y., Zhang J. (2022). The highly conserved RNA-binding specificity of nucleocapsid protein facilitates the identification of drugs with broad anti-coronavirus activity. Comput. Struct. Biotechnol. J..

[22] Ye Q., Lu S., Corbett K.D. (2021). Structural Basis for SARS-CoV-2 Nucleocapsid Protein Recognition by Single-Domain Antibodies. Front. Immunol..

[23] Lopandić Z., Protić-Rosić I., Todorović A., Glamočlija S., Gnjatović M., Ćujic D., Gavrović-Jankulović M. (2021). IgM and IgG Immunoreactivity of SARS-CoV-2 Recombinant M Protein. Int. J. Mol. Sci..

[24] Waeckerle-Men Y., Uetz-von Allmen E., Gander B., Scandella E., Schlosser E., Schmidtke G., Merkle H.P., Groettrup M. (2006). Encapsulation of proteins and peptides into biodegradable poly(D,L-lactide-co-glycolide) microspheres prolongs and enhances antigen presentation by human dendritic cells. Vaccine.

[25] Shastri P.N., Kim M.-C., Quan F.-S., D’Souza M.J., Kang S.-M. (2012). Immunogenicity and protection of oral influenza vaccines formulated into microparticles. J. Pharm. Sci..

[26] Gomes K.B., Menon I., Bagwe P., Bajaj L., Kang S.-M., D’Souza M.J. (2022). Enhanced Immunogenicity of an Influenza Ectodomain Matrix-2 Protein Virus-like Particle (M2e VLP) Using Polymeric Microparticles for Vaccine Delivery. Viruses.

[27] Joshi V.B., Geary S.M., Salem A.K. (2012). Biodegradable Particles as Vaccine Delivery Systems: Size Matters. AAPS J..

[28] Behzadi S., Serpooshan V., Tao W., Hamaly M.A., Alkawareek M.Y., Dreaden E.C., Brown D., Alkilany A.M., Farokhzad O.C., Mahmoudi M. (2017). Cellular uptake of nanoparticles: Journey inside the cell. Chem. Soc. Rev..

[29] Gregory A., Williamson D., Titball R. (2013). Vaccine delivery using nanoparticles. Front. Cell. Infect. Microbiol..

[30] He Y., Park K. (2016). Effects of the Microparticle Shape on Cellular Uptake. Mol. Pharm..

[31] Sun H.-F., Pollock K., Brewer J. (2005). Effects of PLGA microparticles on antigen presentation of bone marrow-derived dendritic cells in vitro. Chin. J. Biomed. Eng..

[32] Snapper C.M. (2018). Distinct Immunologic Properties of Soluble Versus Particulate Antigens. Front. Immunol..

[33] Menon I., Moo Kang S., D’Souza M. (2022). Nanoparticle formulation of the fusion protein virus like particles of respiratory syncytial virus stimulates enhanced in vitro antigen presentation and autophagy. Int. J. Pharm..

[34] Kale A., Joshi D., Menon I., Bagwe P., Patil S., Vijayanand S., Gomes K.B., D’Souza M. (2022). Novel microparticulate Zika vaccine induces a significant immune response in a preclinical murine model after intramuscular administration. Int. J. Pharm..

[35] Vijayanand S., Patil S., Joshi D., Menon I., Braz Gomes K., Kale A., Bagwe P., Yacoub S., Uddin M.N., D’Souza M.J. (2022). Microneedle Delivery of an Adjuvanted Microparticulate Vaccine Induces High Antibody Levels in Mice Vaccinated against Coronavirus. Vaccines.

[36] Escobar-García J.D., Prieto C., Pardo-Figuerez M., Lagaron J.M. (2021). Room Temperature Nanoencapsulation of Bioactive Eicosapentaenoic Acid Rich Oil within Whey Protein Microparticles. Nanomaterials.

[37] Thomas H., Fries F., Gmelch M., Bärschneider T., Kroll M., Vavaleskou T., Reineke S. (2021). Purely Organic Microparticles Showing Ultralong Room Temperature Phosphorescence. ACS Omega.

[38] Kaplan I., Yüksel H., Evliyaoğlu O., Basarali M.K., Toprak G., Çolpan L., Şen V. (2015). Effects of Storage Temperature and Time on Stability of Serum Tacrolimus and Cyclosporine A Levels in Whole Blood by LC-MS/MS. Int. J. Anal. Chem..

[39] Hines D.J., Kaplan D.L. (2013). Poly (lactic-co-glycolic acid) controlled release systems: Experimental and modeling insights. Crit. Rev. Ther. Drug Carrier Syst..

[40] Lengyel M., Kállai-Szabó N., Antal V., Laki A.J., Antal I. (2019). Microparticles, Microspheres, and Microcapsules for Advanced Drug Delivery. Sci. Pharm..

[41] Liang Z., Zhu H., Wang X., Jing B., Li Z., Xia X., Sun H., Yang Y., Zhang W., Shi L. (2020). Adjuvants for Coronavirus Vaccines. Front. Immunol..

[42] Adjuvants and Vaccines|Vaccine Safety|CDC 2020. https://www.cdc.gov/vaccinesafety/concerns/adjuvants.html.

[43] Alhydrogel^®^ Adjuvant 2%. InvivoGen 2016. https://www.invivogen.com/alhydrogel.

[44] Brewer J.M. (2006). (How) do Aluminium Adjuvants Work?. Immunol. Lett..

[45] He P., Zou Y., Hu Z. (2015). Advances in aluminum hydroxide-based adjuvant research and its mechanism. Hum. Vaccin. Immunother..

[46] Nian X., Zhang J., Deng T., Liu J., Gong Z., Lv C., Yao L., Li J., Huang S., Yang X. (2021). AddaVax Formulated with PolyI:C as a Potential Adjuvant of MDCK-based Influenza Vaccine Enhances Local, Cellular, and Antibody Protective Immune Response in Mice. AAPS PharmSciTech.

[47] AddaVax^TM^. InvivoGen 2016. https://www.invivogen.com/addavax.

[48] Wilkins A.L., Kazmin D., Napolitani G., Clutterbuck E.A., Pulendran B., Siegrist C.A., Pollard A.J. (2017). AS03- and MF59-Adjuvanted Influenza Vaccines in Children. Front. Immunol..

[49] Joshi D., Gala R.P., Uddin M.N., D’Souza M.J. (2021). Novel ablative laser mediated transdermal immunization for microparticulate measles vaccine. Int. J. Pharm..

[50] Gala R.P., Zaman R.U., D’Souza M.J., Zughaier S.M. (2018). Novel Whole-Cell Inactivated Neisseria Gonorrhoeae Microparticles as Vaccine Formulation in Microneedle-Based Transdermal Immunization. Vaccines.

[51] Joshi D., Chbib C., Uddin M.N., D’Souza M.J. (2021). Evaluation of Microparticulate (S)-4,5-Dihydroxy-2,3-pentanedione (DPD) as a Potential Vaccine Adjuvant. AAPS J..

[52] Braz Gomes K., D’Sa S., Allotey-Babington G.L., Kang S.-M., D’Souza M.J. (2021). Transdermal Vaccination with the Matrix-2 Protein Virus-like Particle (M2e VLP) Induces Immunity in Mice against Influenza A Virus. Vaccines.

[53] Samimi S., Maghsoudnia N., Eftekhari R.B., Dorkoosh F., Mohapatra S.S., Ranjan S., Dasgupta N., Mishra R.K., Thomas S. (2019). Chapter 3—Lipid-Based Nanoparticles for Drug Delivery Systems. Characterization and Biology of Nanomaterials for Drug Delivery.

[54] Uehara E.U., Shida B.D., de Brito C.A. (2015). Role of nitric oxide in immune responses against viruses: Beyond microbicidal activity. Inflamm. Res..

[55] Wink D.A., Hines H.B., Cheng R.Y., Switzer C.H., Flores-Santana W., Vitek M.P., Ridnour L.A., Colton C.A. (2011). Nitric oxide and redox mechanisms in the immune response. J. Leukoc. Biol..

[56] Bogdan C., Röllinghoff M., Diefenbach A. (2000). The role of nitric oxide in innate immunity. Immunol. Rev..

[57] Tripathi P. (2007). Nitric oxide and immune response. Indian J. Biochem. Biophys..

[58] Frontiers|Redefining the Role of Langerhans Cells as Immune Regulators within the Skin n.d. https://www.frontiersin.org/articles/10.3389/fimmu.2017.01941/full.

[59] Mazzini L., Martinuzzi D., Hyseni I., Benincasa L., Molesti E., Casa E., Lapini G., Piu P., Trombetta C.M., Marchi S. (2021). Comparative analyses of SARS-CoV-2 binding (IgG, IgM, IgA) and neutralizing antibodies from human serum samples. J. Immunol. Methods.

[60] Nie J., Li Q., Wu J., Zhao C., Hao H., Liu H., Zhang L., Nie L., Qin H., Wang M. (2020). Quantification of SARS-CoV-2 neutralizing antibody by a pseudotyped virus-based assay. Nat. Protoc..

[61] Sterlin D., Mathian A., Miyara M., Mohr A., Anna F., Claër L., Quentric P., Fadlallah J., Devilliers H., Ghillani P. (2021). IgA dominates the early neutralizing antibody response to SARS-CoV-2. Sci. Transl. Med..

[62] Aleebrahim-Dehkordi E., Molavi B., Mokhtari M., Deravi N., Fathi M., Fazel T., Mohebalizadeh M., Koochaki P., Shobeiri P., Hasanpour-Dehkordi A. (2022). T helper type (Th1/Th2) responses to SARS-CoV-2 and influenza A (H1N1) virus: From cytokines produced to immune responses. Transpl. Immunol..

[63] Carty S.A., Riese M.J., Koretzky G.A., Hoffman R., Benz E.J., Silberstein L.E., Heslop H.E., Weitz J.I., Anastasi J., Salama M.E., Abutalib S.A. (2018). Chapter 21—T-Cell Immunity. Hematology.

[64] Ganneru B., Jogdand H., Daram V.K., Das D., Molugu N.R., Prasad S.D., Kannappa S.V., Ella K.M., Ravikrishnan R., Awasthi A. (2021). Th1 skewed immune response of whole virion inactivated SARS CoV 2 vaccine and its safety evaluation. IScience.

[65] Getahun A., Dahlström J., Wernersson S., Heyman B. (2004). IgG2a-Mediated Enhancement of Antibody and T Cell Responses and Its Relation to Inhibitory and Activating Fcγ Receptors1. J. Immunol..

